# Is It Time for Population-Based Screening for Transthyretin Cardiac Amyloidosis?

**DOI:** 10.1016/j.jacadv.2024.101233

**Published:** 2024-09-05

**Authors:** Parag Goyal, Daniel M. McDonald

**Affiliations:** a Cardiac Amyloidosis Program, Department of Medicine, Weill Cornell Medicine, New York, New York, USA; b Program for the Care and Study of the Aging Heart, Weill Cornell Medicine, New York, New York, USA

**Keywords:** cardiac amyloidosis, heart failure

**Figure:**
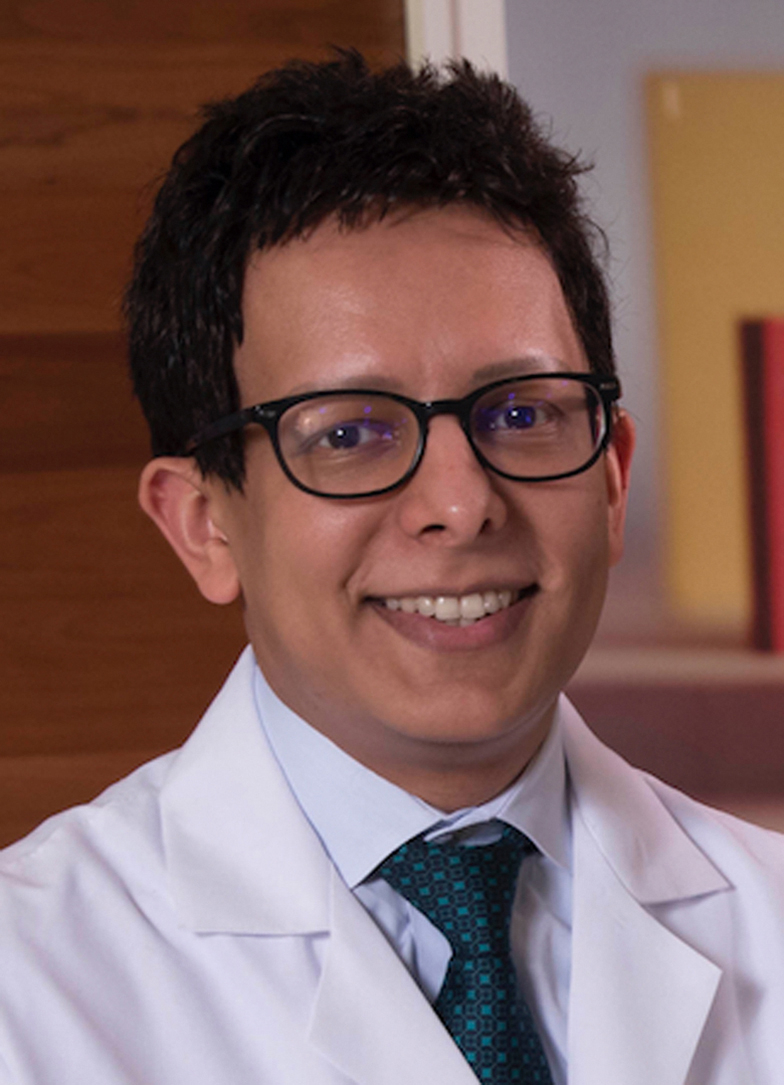

In this issue of *JACC: Advances*, Tini et al^1^ compared outcomes in patients diagnosed with cardiac amyloidosis before and after the introduction of the now widely used noninvasive diagnostic algorithm for transthyretin (TTR) amyloidosis. This retrospective study of 887 adults with cardiac amyloidosis showed that those diagnosed after 2016 had less severe heart failure symptoms and were less likely to have restrictive filling patterns on echocardiography at the time of diagnosis. There was substantial and significantly reduced hazard of death and heart failure hospitalization in the patients diagnosed after 2016 compared to those diagnosed before 2016. The authors emphasize the impact of noninvasive diagnostic algorithms as well as heightened awareness and a proactive approach to a condition that did not have any disease-modifying therapies prior to 2016.^1^

With effective diagnostic approaches and timely initiation of disease-modifying therapy which have led to improved outcomes, population-based screening has emerged as an important consideration. To date, therapeutic options for TTR cardiac amyloidosis can forestall progression but cannot reverse existing pathology. This underscores the importance of diagnosing TTR cardiac amyloidosis as early as possible—with early diagnosis, there is ample opportunity for patients to derive benefit from currently (and soon to be) approved therapies such as TTR stabilizers and silencers. Given the field’s imperative to identify cardiac amyloidosis as early as possible and a broad recognition that TTR cardiac amyloidosis is not as rare as once thought, population-based screening efforts could further improve the health of the population most vulnerable to TTR cardiac amyloidosis—older adults.

There are selected subpopulations of patients in whom TTR cardiac amyloidosis are particularly prevalent. These include patients with heart failure with preserved ejection fraction (prevalence ∼13%-17%)^2^^,^^3^ and those with degenerative aortic stenosis (prevalence ∼16%)^4^; octogenarians, a subpopulation in whom 25% may have amyloid deposition in their heart based on autopsy studies^5^; and those with orthopedic conditions such as carpal tunnel syndrome^6^ and spinal stenosis^7^ which can be caused by deposition of the same amyloid that can affect the heart. Developing and implementing screening approaches in these subpopulations may be a worthwhile endeavor, especially given the high pretest likelihood of such patients which naturally makes any screening process more effective. There may also be value to more broadly screening the population, as there are likely a significant number of at-risk patients outside of these settings. Artificial intelligence has emerged as a potentially fruitful strategy toward this end given its capacity to integrate multiple data points across several domains including demographics, clinical characteristics, biomarkers, and cardiac testing parameters including features from an electrocardiogram and/or echocardiogram. Such an approach could help to identify at-risk patients, who would then undergo the routine diagnostic workup for cardiac amyloidosis including technetium-labeled radionuclide bone scintigraphy to establish a diagnosis.

As the field moves toward such population-based strategies, there are some important considerations related to diagnosis and subsequent management of positive diagnostic testing. First, we need to ensure that our noninvasive approach to diagnosing cardiac amyloidosis (currently via technetium-labeled radionuclide bone scintigraphy) has acceptable diagnostic performance (sensitivity, specificity, negative predictive value, positive predictive value) within a broad population. Seminal work on the diagnostic performance of technetium-labeled radionuclide bone scintigraphy (which led to its formal inclusion in the current diagnostic algorithm for cardiac amyloidosis) was done in a population with a high pretest probability—in those studies, false positives (positive test without evidence of any form of cardiac amyloidosis) were nearly nonexistent.^8^ In a broader population with intermediate (and perhaps even some low) pretest probability, the risk for false positive technetium-labeled radionuclide bone scintigraphy may be elevated. In addition to minimizing false positives, it will be similarly important to minimize false negatives—prior studies have shown that the sensitivity of technetium-labeled radionuclide bone scintigraphy is only 70% even among those with a high pretest probability.^8^ This risk may be particularly problematic for those with early disease, the very focus of population-based screening. Both misdiagnosis (false positives) and missed diagnoses (false negatives) would have implications on patient care and health care costs. This underscores the importance of developing improved diagnostic modalities, an area under substantive development over the past few years with great promise for improving the effectiveness of population-based screening processes.^9^

Second, we need to ensure that population-based approaches reach all populations, including those who are underrepresented. This is especially relevant given the increased risk of cardiac amyloidosis observed in persons of Afro-Caribbean descent, which is attributed to a carrier status of the V142I variant in the TTR gene that is estimated to occur in nearly 3 to 4% of persons who self-identify as African American or Black in the United States.^10^ Challenges to genetic screening efforts for this subpopulation have previously been enumerated—these include referral bias, health literacy, and medical distrust among others,^11^ and are likely to be operative in the broader sense of population-based screening for cardiac amyloidosis. There is an unfortunate history whereby efforts intended to improve the health of a population have inadvertently exacerbated health care disparities.^12^ Strategies must accordingly be incorporated to prevent this and to ensure that the population that stands to derive the greatest benefit is prioritized.

Third, we need to promote the notion that patients with positive diagnostic testing for cardiac amyloidosis can have or develop additional etiologies of a heart failure syndrome. For example, coronary artery disease and valvular disease become increasingly common with age and are more common as causes of heart failure than cardiac amyloidosis. Accordingly, even when testing is positive and a diagnosis of cardiac amyloidosis is established, it is important for clinicians to continue to consider these traditional etiologies of heart failure which could additionally contribute to the underlying substrate. While disease-modifying therapy for cardiac amyloidosis would be indicated regardless, such patients may warrant additional treatment (such as revascularization or a valvular intervention). In short, a positive technetium-labeled radionuclide bone scintigraphy should not preclude clinicians from seeking additional testing and/or consideration and treatment of additional etiologies when there are features suggesting their presence. Moving forward, integrating multimodal imaging with capacity for improved tissue characterization into the diagnostic algorithm of cardiac amyloidosis could be helpful for increased precision of heart failure phenotyping.

The authors Tini et al^1^ have drawn attention to impressive progress made in the field of cardiac amyloidosis since 2016. Based on these data, it is likely that broader screening efforts that incorporate the noninvasive diagnostic algorithm for TTR amyloidosis will identify an increasing number of previously unrecognized cardiac amyloidosis, and earlier in the disease state than during the prior era. However, as outlined, there remain some important issues to consider as these efforts take shape.^1^ Given the implications of these issues, it may be important to link screening efforts with dedicated programs and specialists that have the expertise in cardiac amyloidosis to help manage the subtleties of diagnosis. Their expertise will be critical to provide the right diagnosis and optimize management. Population-based screening is a natural next step for the field—but it is imperative that we do not replace missed diagnosis with misdiagnosis.

## Funding support and author disclosures

Dr Goyal is supported by 10.13039/100000049National Institute on Aging grants K76AG064428, R21AG077092, R01AG085420, and a U.S. Deprescribing Research Pilot Grant funded by the 10.13039/100000049National Institute on Aging (R24AG064025); has received consulting fees from Agepha Pharma, Akros Pharma, Axon therapies, Bayer HealthCare Pharmaceuticals, and Sensorum Health; and has received personal fees for medicolegal consulting and expert testimony related to heart failure. Dr McDonald has reported that he has no relationships relevant to the contents of this paper to disclose.
